# Co-Exposure to Aflatoxin B1 and Patulin Induces Hepatic Injury in Mice and HepG2 Cells by Activating Oxidative Stress and Apoptosis

**DOI:** 10.3390/toxins18050197

**Published:** 2026-04-23

**Authors:** Yaqian Liu, Shimin Lei, Yixuan Peng, Yuan Li, Xingxiang Chen, Xinyi Xu, Sichao Mao

**Affiliations:** 1 College of Veterinary Medicine, Nanjing Agricultural University, Nanjing 210095, China; 2023107100@stu.njau.edu.cn (Y.L.); 2023807178@stu.njau.edu.cn (S.L.); 2024107098@stu.njau.edu.cn (Y.P.); 2024807191@stu.njau.edu.cn (Y.L.); cxx@njau.edu.cn (X.C.); 2022057@njau.edu.cn (X.X.); 2 Institute of Animal Nutrition Health, Nanjing Agricultural University, Nanjing 210095, China; 3 MOE Joint International Research, Nanjing Agricultural University, Nanjing 210095, China

**Keywords:** aflatoxin B1, patulin, no-observed adverse effect levels, hepatotoxicity, oxidative stress, caspase-dependent apoptosis

## Abstract

Aflatoxin B1 (AFB1) and patulin (PAT) are prevalent foodborne mycotoxins with hepatotoxic potential, but the hepatic effects of combined exposure remain largely unclear. This study investigated the hepatotoxic consequences of co-exposure to AFB1 and PAT using no-observed adverse effect levels (NOAELs) in C57BL/6 mice and low-cytotoxic concentrations in HepG2 cells selected by viability screening. Mice and cells were assigned to four groups: control, AFB1, PAT and AFB1 + PAT. Exposure to either toxin individually did not cause evident liver injury, whereas co-exposure significantly elevated alanine aminotransferase (ALT) and aspartate aminotransferase (AST) activities, reduced liver index, and induced clear histopathological alterations. Co-exposure markedly aggravated oxidative stress, characterized by increased reactive oxygen species (ROS) and malondialdehyde (MDA) and decreased superoxide dismutase (SOD). In parallel, the levels of interleukin-6 (IL-6), interleukin-1 beta (IL-1β), and tumor necrosis factor-alpha (TNF-α) were elevated, together with the early fibrosis-related markers alpha-smooth muscle actin (α-SMA) and vimentin. The apoptotic response was characterized by increased Bcl-2-associated X protein (Bax) and reduced B-cell lymphoma-2 (Bcl-2), together with cysteine-dependent aspartate-specific protease-3 (caspase-3) activation. These findings indicate that co-exposure to AFB1 and PAT elicits hepatotoxicity through amplified oxidative stress, inflammation, and caspase-dependent apoptosis, supporting the need to further consider mycotoxin co-exposure in toxicological evaluation.

## 1. Introduction

Mycotoxins are toxic fungal metabolites generated during the growth, harvest, transportation, and storage of agricultural commodities [1,2,3,4]. These contaminants enter the food chain via grains, fruits, nuts, and other crops [5]. Their widespread occurrence has made mycotoxin exposure a major global food safety concern, threatening both human health and animal production [6,7]. Some mycotoxins are associated with multi-organ injuries involving the liver, kidney, immune system, and nervous system, and they are also linked to carcinogenic and teratogenic risks under specific exposure conditions [8,9].

Aflatoxin B1 (AFB1) has long been a significant concern due to its high toxicity and well-established carcinogenicity. It is primarily produced by *Aspergillus flavus* and *Aspergillus parasiticus*, frequently detected in staple crops such as maize, peanuts, and rice [10,11,12]. The International Agency for Research on Cancer (IARC) has assigned AFB1 to Group 1 of its carcinogen classification scheme [13,14,15]. After entering hepatocytes, AFB1 is bioactivated by cytochrome P450 enzymes to yield aflatoxin B1-8,9-epoxide, a highly reactive intermediate capable of binding DNA and initiating mutagenic events associated with hepatocarcinogenesis [16,17,18].

Beyond these well-characterized genotoxic properties, AFB1 is frequently associated with oxidative stress and mitochondria-related injury signaling. Earlier reports have demonstrated that AFB1 exposure promotes reactive oxygen species (ROS) accumulation and disrupts redox homeostasis, along with pro-apoptotic alterations including Bcl-2-associated X protein (Bax)/B-cell lymphoma-2 (Bcl-2) imbalance and cysteine-dependent aspartate-specific protease-3 (caspase-3) activation [19,20,21,22].

Patulin (PAT) is another widely encountered mycotoxin, mainly synthesized by *Penicillium expansum* and related fungal species [23,24]. It is commonly identified in apples, pears, hawthorns, and their processed products, including juices and jams [25,26]. PAT is classified by IARC as Group 3 (not classifiable as to its carcinogenicity to humans) and has been associated with immunosuppression, neurotoxicity, and gastrointestinal injury [27,28]. PAT can react with intracellular thiol-containing compounds and deplete glutathione (GSH), thereby weakening antioxidant defenses and promoting ROS accumulation [29,30]. PAT exposure has been mechanistically linked to the collapse of mitochondrial membrane potential, an imbalance in pro- and anti-apoptotic Bcl-2 family proteins, accompanied by caspase-3 activation, collectively triggering the mitochondrial intrinsic apoptotic program [31,32,33].

In practical settings, food and feed are rarely contaminated by a single mycotoxin [34]. Instead, multiple mycotoxins frequently co-occur during production, processing, and storage, representing a substantial but often underestimated food safety risk [35,36]. According to the 2025 revision of the General Standard for Contaminants and Toxins in Food and Feed, maximum limits for AFB1 are typically set between 5–20 µg/kg for different food categories, while the maximum limit for PAT in fruit juices and related products is 50 µg/kg [37]. However, despite these stringent international limits, co-occurrence of mycotoxins such as AFB1 and PAT remains common in agricultural commodities and feeds [12,38]. Increasing evidence suggested that multiple mycotoxins may exert additive or even interactive toxicity through shared or overlapping pathways, thereby amplifying damage to the host [39,40]. Given that both AFB1 and PAT are linked to oxidative stress and apoptosis-related signaling [41,42,43], it remains insufficiently characterized whether their co-occurrence at no-observed adverse effect levels (NOAELs) amplifies hepatotoxicity and which key biological processes are involved. AFB1 and PAT were selected because both are important foodborne mycotoxins and may contribute to mixed dietary exposure. This study therefore evaluated the toxicological relevance of their co-exposure.

Accordingly, the present study was designed to determine whether co-exposure to AFB1 and PAT at NOAEL-related doses in mice and low-cytotoxic concentrations in HepG2 cells induces greater hepatotoxicity than individual exposure. The involvement of oxidative stress, inflammatory activation, and caspase-dependent apoptosis was further evaluated in parallel in vivo (C57BL/6 mice) and in vitro (HepG2 cells) models, thereby providing critical experimental data for risk assessment of multi-mycotoxin co-exposure.

## 2. Results

### 2.1. Co-Exposure to AFB1 and PAT Induced Liver Injury, Oxidative Stress, and Histopathological Alterations in Mice

**Figure 1:**
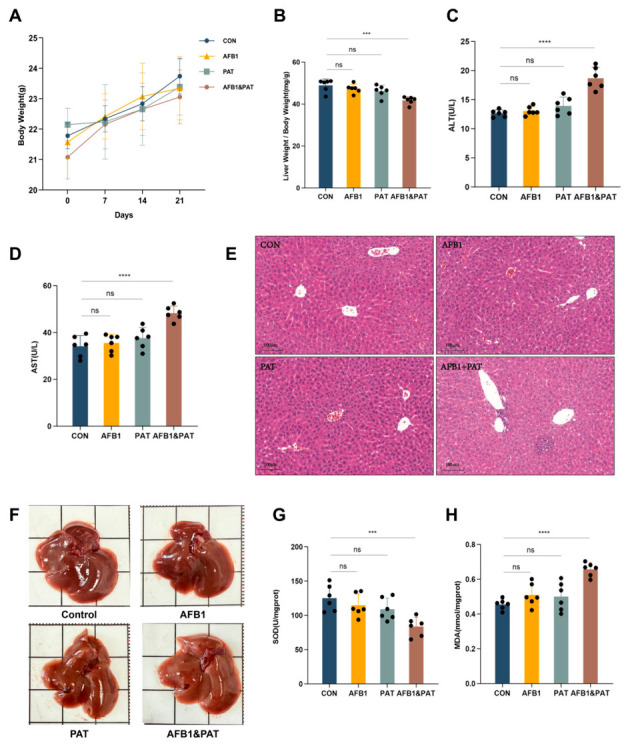
Co-exposure to AFB1 and PAT induced liver dysfunction, oxidative stress, and histopathological alterations in mice. (**A**) Body weight of mice. (**B**) Liver organ index. (**C**,**D**) Serum ALT and AST levels. (**E**) H&E staining of liver sections (100×). (**F**) Gross morphology of the liver. (**G**,**H**) Hepatic SOD and MDA activity. All data are presented as mean ± SD (*n* = 6). Statistics were analyzed by one-way ANOVA followed by Tukey’s test, and AFB1 × PAT interaction effects were additionally evaluated by two-way ANOVA as described in the Results Section. ns, not significant (*p* > 0.05). *** *p* < 0.001; **** *p* < 0.0001.

Following a 21-day oral gavage, liver injury markers, redox indices, and histopathological changes were assessed. AFB1 or PAT individually did not significantly affect the liver index, whereas a significant reduction was observed in the co-exposure group (*p* < 0.001, Figure 1B). Body weight showed a decreasing trend, although no significant difference was detected (Figure 1A). Single-toxin exposure did not significantly alter serum alanine aminotransferase (ALT) or aspartate aminotransferase (AST), whereas co-exposure markedly elevated both enzymes (*p* < 0.0001, Figure 1C,D).

Grossly, livers from the control and single-toxin groups appeared normal (dark-red and smooth), whereas those from the co-exposed group were yellow-red with diffuse surface spots (Figure 1F). Histological examination showed that the control group maintained an intact hepatic structure, whereas hepatocyte swelling, disorganized hepatic cords, and inflammatory cell infiltration were observed in the co-exposure group (Figure 1E). Accompanying these structural changes, the co-exposure group showed significantly higher malondialdehyde (MDA) levels and a marked reduction in superoxide dismutase (SOD) activity (*p* < 0.001, Figure 1G,H). In two-way ANOVA, the AFB1 × PAT interaction term was significant for serum ALT, AST, and MDA, but not for liver index or hepatic SOD activity.

### 2.2. Co-Exposure to AFB1 and PAT Induced Cytotoxicity in HepG2 Cells

**Figure 2:**
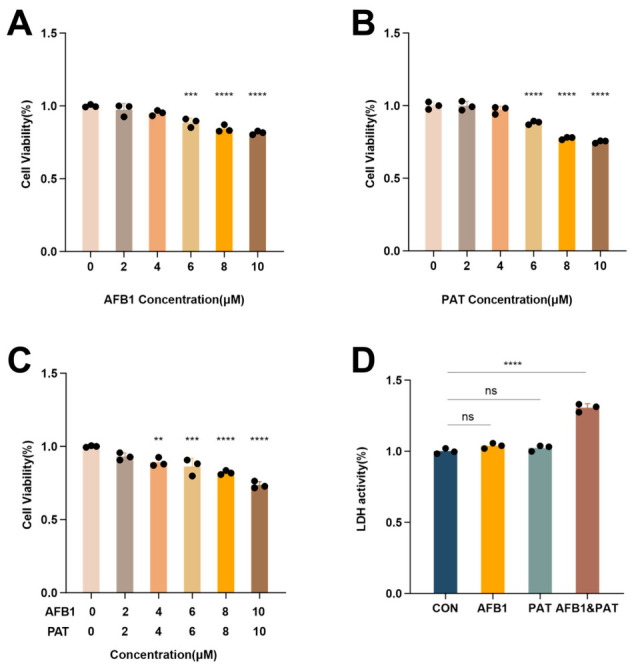
Co-exposure to AFB1 and PAT induced cytotoxicity in HepG2 cells. (**A**,**B**) Cell viability of HepG2 cells treated with different concentrations of AFB1 or PAT for 24 h. (**C**) Cell viability after AFB1 and PAT co-exposure. (**D**) LDH release in HepG2 cells after treatment with AFB1 (4 μM), PAT (4 μM), or their combination. All data are presented as mean ± SD (*n* = 6). Statistics were analyzed by one-way ANOVA followed by Tukey’s test, and AFB1 × PAT interaction effects were additionally evaluated by two-way ANOVA as described in the Results Section. ns, not significant (*p* > 0.05). ** *p* < 0.01; *** *p* < 0.001; **** *p* < 0.0001.

CCK-8 screening showed that AFB1 and PAT each maintained HepG2 viability above 90% at 0–4 μM, whereas 6–10 μM caused significant cytotoxicity (Figure 2A,B; *p* < 0.0001 for PAT). Accordingly, 4 μM of each toxin was selected for subsequent experiments. HepG2 cells were subsequently exposed to AFB1 and PAT in combination. Co-exposure to AFB1 (4 μM) and PAT (4 μM) significantly reduced cell viability versus controls (*p* < 0.01), with cytotoxicity increasing at higher concentrations (Figure 2C). Lactate dehydrogenase (LDH) release was unaffected by either toxin alone but was markedly elevated by combined exposure (*p* < 0.0001, Figure 2D). In two-way ANOVA, the AFB1 × PAT interaction term was significant for LDH release.

### 2.3. Co-Exposure to AFB1 and PAT Induced Oxidative Stress in HepG2 Cells

**Figure 3:**
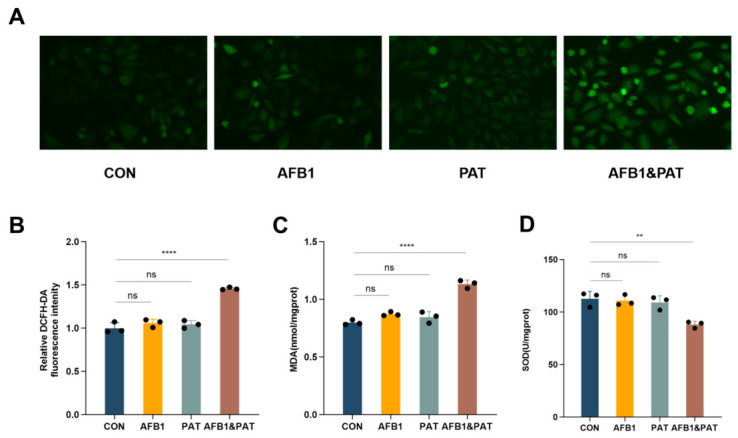
Co-exposure to AFB1 and PAT induced oxidative stress in HepG2 cells. (**A**) Representative DCFH-DA fluorescence images. (**B**) Quantification of intracellular ROS fluorescence intensity. (**C**,**D**) Intracellular SOD and MDA activity. All data are presented as mean ± SD (*n* = 3). Statistics were analyzed by one-way ANOVA followed by Tukey’s test, and AFB1 × PAT interaction effects were additionally evaluated by two-way ANOVA as described in the Results Section. ns, not significant (*p* > 0.05). ** *p* < 0.01; **** *p* < 0.0001.

To ascertain whether in vivo oxidative stress findings were recapitulated in vitro, intracellular ROS, MDA, and SOD were assessed in HepG2 cells. Treatment with AFB1 or PAT individually did not notably increase ROS accumulation, whereas co-exposure markedly enhanced intracellular ROS fluorescence intensity (*p* < 0.0001, Figure 3A,B). Combined exposure also significantly elevated MDA content (*p* < 0.01, Figure 3C) and reduced SOD enzymatic activity (*p* < 0.0001, Figure 3D), consistent with the in vivo findings. The AFB1 × PAT interaction term was significant for ROS, MDA, and SOD in two-way ANOVA.

### 2.4. Co-Exposure to AFB1 and PAT Activated Inflammation and Induced Early Fibrotic Marker Expression

**Figure 4:**
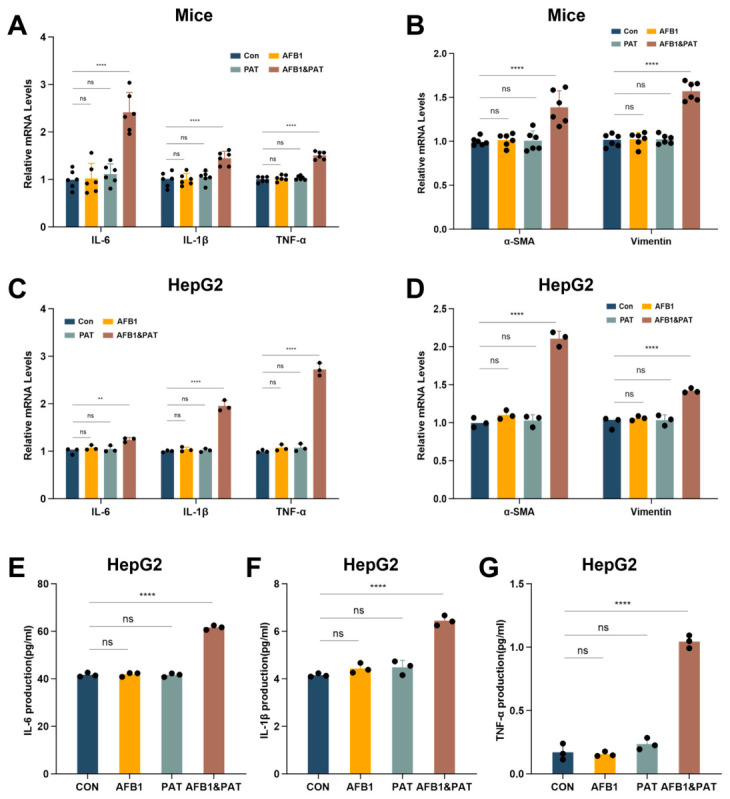
Co-exposure to AFB1 and PAT activated inflammatory responses and induced early fibrotic marker expression. (**A**–**D**) mRNA expression of IL-6, IL-1β, TNF-α, α-SMA and vimentin in mouse liver tissues and HepG2 cells. (**E**–**G**) Protein levels of inflammatory cytokines in HepG2 cell culture supernatants measured by ELISA. All data are presented as mean ± SD (*n* = 3 in HepG2 cells; *n* = 6 in mice). Statistics were analyzed by one-way ANOVA followed by Tukey’s test, and AFB1 × PAT interaction effects were additionally evaluated by two-way ANOVA as described in the Results Section. ns, not significant (*p* > 0.05). ** *p* < 0.01; **** *p* < 0.0001.

In mouse liver tissues, AFB1 or PAT individually did not significantly modulate the mRNA levels of interleukin-1 beta (IL-1β), interleukin-6 (IL-6) or tumor necrosis factor alpha (TNF-α). In contrast, co-exposure resulted in a robust upregulation of these pro-inflammatory cytokines at the transcriptional level (*p* < 0.0001) (Figure 4A). Additionally, the mRNA levels of the early fibrotic markers (alpha-smooth muscle actin (α-SMA) and vimentin) were markedly elevated in mice receiving the combined treatment (*p* < 0.0001) (Figure 4B). A similar pattern was observed in HepG2 cells (Figure 4C,D), and enzyme-linked immunosorbent assay (ELISA) further showed markedly elevated protein secreted concentrations of pro-inflammatory cytokines in conditioned medium (*p* < 0.0001, Figure 4E–G). In mouse liver tissues, the AFB1 × PAT interaction term was significant for inflammation and fibrosis genes in two-way ANOVA. In HepG2 cells, the interaction term was significant for all endpoints except IL-6 mRNA expression.

### 2.5. Co-Exposure to AFB1 and PAT Induced Hepatocyte Apoptosis In Vivo

**Figure 5:**
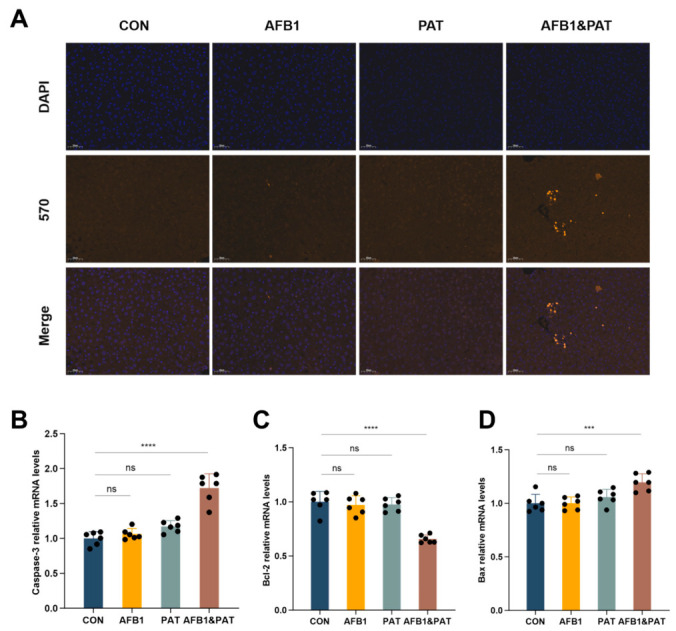
Co-exposure to AFB1 and PAT induced hepatocyte apoptosis in vivo. (**A**) Representative TUNEL staining of liver sections, with nuclei counterstained by DAPI. (**B**–**D**) mRNA expression levels of Bax, Bcl-2, and caspase-3 in mouse liver tissues. All data are presented as mean ± SD (*n* = 6). Statistics were analyzed by one-way ANOVA followed by Tukey’s test, and AFB1 × PAT interaction effects were additionally evaluated by two-way ANOVA as described in the Results Section. ns, not significant (*p* > 0.05). *** *p* < 0.001; **** *p* < 0.0001.

Hepatocyte apoptosis was evaluated in mouse liver tissues. Terminal deoxynucleotidyl transferase dUTP nick-end labeling (TUNEL) staining revealed sparse positive cells in control and single-toxin groups, whereas co-exposure markedly increased TUNEL-positive signals with discernible nuclear morphological changes in a subset of hepatocytes (Figure 5A). Co-exposure also significantly upregulated transcriptional levels of the apoptosis-related genes Bax and caspase-3 (*p* < 0.0001), accompanied by reciprocal suppression of the cell survival-related protein Bcl-2 (*p* < 0.001) relative to control and single-toxin groups (Figure 5B–D). The AFB1 × PAT interaction term was significant for Bax, Bcl-2, and caspase-3 in two-way ANOVA.

### 2.6. The Pan-Caspase Inhibitor Z-VAD-FMK Attenuated AFB1 and PAT-Induced Apoptosis in HepG2 Cells

**Figure 6:**
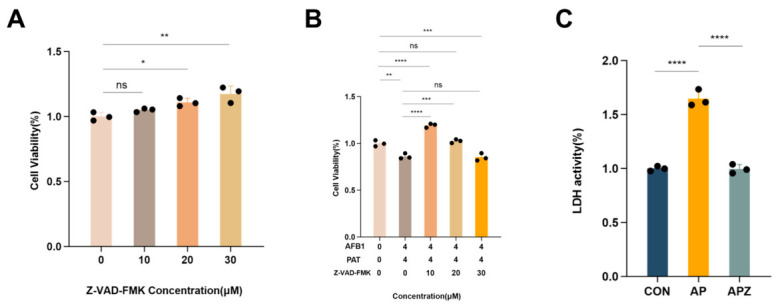
Pan-caspase inhibitor Z-VAD-FMK markedly ameliorated AFB1 and PAT-induced cytotoxicity in HepG2 cells. (**A**) Cell viability after treatment with Z-VAD-FMK. (**B**) Cell viability after Z-VAD-FMK pretreatment during combined AFB1 and PAT exposure. (**C**) LDH activity in the control (CON), AFB1 + PAT (AP), and AFB1 + PAT + Z-VAD-FMK (APZ) groups. Data are presented as mean ± SD (*n* = 3). Statistics were analyzed by one-way ANOVA followed by Tukey’s test. ns, not significant (*p* > 0.05). * *p* < 0.05; ** *p* < 0.01; *** *p* < 0.001; **** *p* < 0.0001.

HepG2 cells were pretreated with the pan-caspase inhibitor Z-VAD-FMK for 1 h before co-exposure. Z-VAD-FMK showed no significant effect on cell viability at a concentration of 10–30 μM; a modest yet statistically discernible elevation in cell viability was observed at 20 μM (*p* < 0.05) and 30 μM (*p* < 0.01, Figure 6A). Co-exposure significantly reduced viability (*p* < 0.01), which was fully restored by 10 μM Z-VAD-FMK pretreatment (*p* < 0.0001, viability > 100%), and partially ameliorated at 20 μM (*p* < 0.001). However, pretreatment with 30 μM Z-VAD-FMK further decreased viability (*p* < 0.001, Figure 6B). Therefore, 10 μM Z-VAD-FMK was selected for subsequent analyses. Co-exposure also elevated LDH release (*p* < 0.0001), which was markedly reduced by Z-VAD-FMK pretreatment (*p* < 0.0001, Figure 6C).

**Figure 7:**
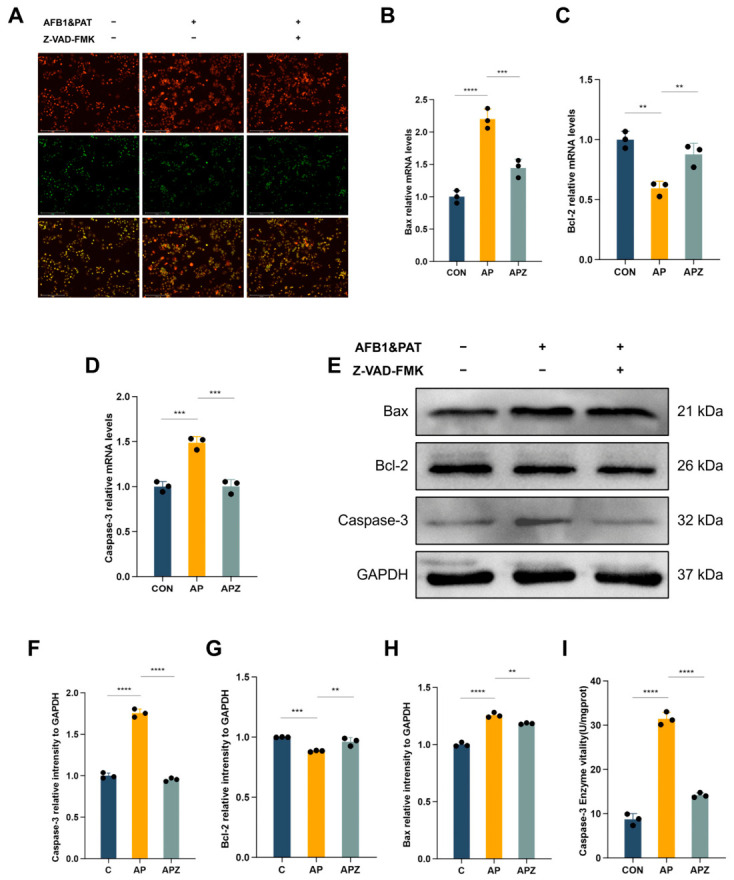
Z-VAD-FMK suppresses caspase-dependent apoptosis induced by combined AFB1 and PAT exposure. (**A**) AO/EB staining of HepG2 cells. (**B**–**D**) mRNA expression levels of Bax, Bcl-2, and caspase-3. (**E**–**H**) Relative protein expression levels of Bax, Bcl-2, caspase-3, and GAPDH. (**I**) caspase-3 activity assay. The groups were defined as follows: control (C), AFB1 + PAT (AP), and AFB1 + PAT + Z-VAD-FMK (APZ). Samples were derived from parallel experiments, and the gels were processed in parallel. Data are presented as mean ± SD (*n* = 3). Statistics were analyzed by one-way ANOVA followed by Tukey’s test. ** *p* < 0.01; *** *p* < 0.001; **** *p* < 0.0001.

AO/EB staining showed a pronounced increase in apoptotic cells after co-exposure, which was substantially reduced by Z-VAD-FMK pretreatment (Figure 7A). At the molecular level, co-exposure significantly upregulated Bax (*p* < 0.0001) and caspase-3 (*p* < 0.001) mRNA while reducing Bcl-2 expression (*p* < 0.01); Z-VAD-FMK pretreatment significantly attenuated Bax and Caspase-3 upregulation (*p* < 0.001 and *p* < 0.01, respectively) and partially restored Bcl-2 (*p* < 0.01, Figure 7B–D). Western blot (WB) analysis showed the same trend at the protein level (Figure 7E–H). Co-exposure-induced caspase-3 activity was also significantly suppressed by Z-VAD-FMK pretreatment (*p* < 0.0001), albeit not to basal levels (Figure 7I).

## 3. Discussion

Aflatoxin B1 (AFB1) and patulin (PAT) are produced by fungal contamination during food storage and processing, particularly under unfavorable conditions, and are detectable across a wide range of agricultural commodities and derived products [1,3]. Under realistic exposure scenarios, co-contamination with multiple mycotoxins is more common [38,39], yet evidence on the joint effects of AFB1 and PAT under the selected dose conditions remains limited. At higher doses, both toxins individually trigger oxidative stress and inflammatory responses that promote hepatocellular damage [9,20,44]. Based on these findings, the liver was selected as the main target organ, and the hepatotoxicity of co-exposure to AFB1 and PAT under the present experimental conditions was evaluated using C57BL/6 mice and HepG2 cells.

In an in vivo model, AFB1 (10 μg/kg) and PAT (30 μg/kg) were selected to approximate dietary-relevant exposures and to test whether the hepatotoxic cascade could be initiated under the selected co-exposure conditions. Regulatory frameworks also reflect that AFB1 and PAT in foods are typically controlled at the μg/kg level [37]. Mechanistically, the relatively high constitutive activity of hepatic glutathione S-transferase A3 (mGSTA3) in mice has been linked to an intrinsic resistance to AFB1-induced hepatotoxicity [45], and subchronic exposure to AFB1 at 10 μg/kg for 28 days has been reported to elicit immunological alterations rather than overt hepatic necrosis or functional failure [46]; even at a higher dose of 44 μg/kg, serum liver enzymes were not markedly changed [47]. Established toxicological reference values, corroborated by independent dose–response studies, converge on a NOAEL of approximately 43 μg/kg for PAT [48,49], and repeated exposure at 80 μg/kg for 28 days has not been associated with clear changes in liver weight or gross pathology [50], supporting 30 μg/kg as a range generally considered without overt hepatotoxicity. In vitro, 4 μM AFB1 and 4 μM PAT were selected based on preliminary viability screening; at these levels, exposure to either mycotoxin alone maintained cell viability above 90%, thereby establishing a low-cytotoxic experimental condition for evaluating the effects of co-exposure. These concentrations were intended to provide an in vitro mechanistic window rather than to reproduce measured human biomonitoring levels.

Under these dose conditions, exposure to AFB1 or PAT individually did not produce overt abnormalities in liver function or histopathology. In contrast, co-exposure led to increased serum ALT and AST, reduced liver indices, and prominent morphological alterations, including hepatocellular swelling, disorganization of hepatic architecture, and inflammatory cell infiltration. These findings indicate that a more pronounced hepatotoxic phenotype can emerge even when the effects of individual toxins remain modest. Similar patterns of amplified liver injury following co-exposure have also been reported for other mycotoxin combinations; for example, Sun et al. observed elevated liver enzyme activities in mice exposed to combined AFB1 and DON [51]. According to Slinker, interaction in a 2 × 2 design should be evaluated by testing the interaction term in two-way ANOVA [52]. In the present study, the significant AFB1 × PAT interaction terms indicate that, under the tested condition, the combined effects of AFB1 and PAT were greater than would be expected from either toxin alone. However, because only one dose combination was examined, these results support interaction only at the selected condition and do not provide definitive evidence of synergism across different dose ranges. Collectively, these results suggest that co-exposure may exacerbate liver injury by accumulating early stress signals under the present experimental conditions.

In the liver, a highly metabolically active organ, disruption of redox homeostasis together with inflammatory activation is widely recognized as an early process in mycotoxin-associated injury [53]. A pair-feeding experiment in pullets showed that co-contamination with AFB1 and OTA alters oxidative stress indices (e.g., ROS, MDA, and SOD) and reshapes cytokine profiles, indicating that mycotoxin co-exposure can concurrently disrupt antioxidant defenses and inflammatory regulation [54]. In vitro evidence further suggests that co-exposure to AFB1 and DON increases ROS and MDA to a greater extent than single-toxin treatment, thereby imposing a greater oxidative burden [55]. In alignment with this body of evidence, the current experimental data demonstrate that oxidative stress markers and pro-inflammatory mediators did not differ significantly from the control group following low exposure to AFB1 or PAT individually. This likely reflects the capacity of endogenous antioxidant systems to maintain redox balance under mild toxicant challenge. However, co-exposure produced clear and concordant changes in vivo and in vitro, characterized by elevated ROS and MDA levels, reduced SOD activity, and pronounced transcriptional induction of IL-1β, IL-6, and TNF-α involved in the inflammatory response. Compared with either single-toxin exposure, the combined treatment produced broader and more pronounced oxidative stress- and inflammation-related responses, with significant AFB1 × PAT interaction terms observed for several endpoints. Furthermore, early elevation of α-SMA and vimentin suggests that inflammation-associated remodeling signals are engaged and may contribute to subsequent injury progression.

Sustained oxidative stress and inflammation can facilitate the accumulation of apoptotic signaling and activate caspase-3 through the caspase cascade, thereby executing apoptosis [56]. Co-exposure to AFB1 and PAT under the present experimental conditions significantly disrupted the balance of Bcl-2 family members, as evidenced by a reciprocal shift toward Bax predominance and Bcl-2 depletion at the transcript level, along with activation of the downstream effector caspase-3. This pattern is consistent with prior observations in HepG2 cells, where co-exposure of AFB1 and DON amplifies Bax and caspase-3 upregulation while suppressing Bcl-2 [51]. PAT has also been reported to trigger caspase signaling via ROS-related stress [57]. Concordantly, TUNEL labeling of liver sections demonstrated a substantial expansion of apoptosis-committed hepatocytes specifically in the co-exposure group, further supporting the involvement of apoptosis in the injury process. Pan-caspase inhibitor Z-VAD-FMK was applied in vitro; pretreatment attenuated the cytotoxic and apoptotic phenotypes and reduced caspase-3 activity, indicating that apoptosis induced by co-exposure under the selected conditions was largely mediated through the caspase pathway. Similarly, Mao et al. reported that Z-VAD-FMK effectively blocked caspase-3 activation and reduced LDH release and inflammation-related outputs in a DON-induced hepatocyte injury model [58].

However, several limitations of this study warrant attention. Although the four-group data were reanalyzed using a 2 × 2 factorial design and significant AFB1 × PAT interaction terms were identified for multiple endpoints, the study was conducted at only a single dose combination. Therefore, the present data do not allow a full characterization of dose–response relationships or a definitive distinction among additive, synergistic, and antagonistic patterns across different exposure levels. In addition, the in vitro concentrations were selected on the basis of viability screening to establish a low-cytotoxic experimental condition and were not intended to reproduce the much lower internal exposure levels generally reported in human biomonitoring studies. The limited CYP-mediated metabolic capacity of HepG2 cells may lead to underestimation of the metabolism-dependent toxicity of AFB1 in vitro, and more metabolically competent hepatic models are needed to validate these findings. Accordingly, the present findings should not be directly extrapolated to regulatory exposure thresholds without broader dose-range studies and more physiologically relevant models. Furthermore, the upstream signaling pathways underlying these responses were not investigated and represent a direction for future study.

## 4. Conclusions

Compared with individual toxin exposure, co-exposure to AFB1 and PAT elicits a more pronounced hepatic injury response, reflected by impaired liver function indices, enhanced oxidative stress, upregulated inflammatory mediators, and activation of caspase-dependent apoptosis. Given that AFB1 and PAT may contribute to mixed dietary exposure, these findings support the need to further consider mycotoxin co-exposure in toxicological evaluation. Future work is needed to clarify the long-term effects of co-exposure, dose–response relationships, and the key regulatory mechanisms involved, thereby providing a stronger basis for health risk assessment and potential intervention strategies.

## 5. Materials and Methods

### 5.1. Reagents and Antibodies

AFB1 was sourced from Acmec (Shanghai, China). PAT was procured from Macklin (Shanghai, China). Dimethyl sulfoxide (DMSO) was supplied by Sigma-Aldrich (St. Louis, MO, USA). Phenylmethanesulfonyl fluoride (PMSF), radio immunoprecipitation assay (RIPA) lysis buffer, and a BCA protein quantification kit were all purchased from Beyotime Biotechnology (Shanghai, China). The pan-caspase inhibitor Z-VAD-FMK was acquired from SparkJade (Jinan, China). Polyclonal antibodies directed against Bax, Bcl-2, and Caspase-3 were obtained from ABclonal (Wuhan, China). The glyceraldehyde-3-phosphate dehydrogenase (GAPDH) antibody was purchased from Proteintech (Wuhan, China). HRP-conjugated secondary antibody (goat anti-rabbit IgG) was acquired from Abmart (Shanghai, China).

### 5.2. Animal Experimental Design and Sample Collection

Twenty-four male C57BL/6 mice (6 weeks old; 20–22 g body weight) were procured from Yangzhou University (Yangzhou, China). The mice were maintained in a standard environment (23 ± 1 °C; 60 ± 10% relative humidity; 12 h light/dark cycle) with free access to standard chow and water. All experimental procedures involving animals were approved by the Institutional Animal Care and Use Committee of Nanjing Agricultural University (Permit No. SYXK (Su) 2023-0926) and conducted in accordance with the National Institutes of Health (NIH) Guide for the Care and Use of Laboratory Animals. This study was designed, conducted, and reported in compliance with the ARRIVE (Animal Research: Reporting of In Vivo Experiments) guidelines.

Following a one-week acclimatization period, mice were randomly allocated to four groups (*n* = 6 per group): control group, AFB1 (10 μg/kg BW), PAT (30 μg/kg BW), and AFB1 + PAT (10 μg/kg BW AFB1 plus 30 μg/kg BW PAT). All treatments were delivered once daily via oral gavage for 21 days. Mice were euthanized following the final treatment. After blood collection, samples were centrifuged at 3000 rpm for 15 min to separate serum, which was subsequently preserved for biochemical assays. The liver was excised, rinsed with cold saline, and then weighed. The ratio of liver weight to body weight was used to calculate the liver index. Representative liver tissues were immersion-fixed in 4% paraformaldehyde for histopathological and TUNEL analyses. The remaining samples were immediately frozen in liquid nitrogen and stored at −80 °C.

### 5.3. Histopathological and TUNEL Analyses

After fixation in 4% paraformaldehyde, liver tissues were dehydrated in graded ethanol, embedded in paraffin, and sectioned into 4 μm slices. Following hematoxylin and eosin (H&E) staining, histological changes were evaluated and photographed under a light microscope. For apoptosis detection, additional slices were processed with a commercial one-step TUNEL kit (Beyotime, Shanghai, China). TUNEL signals were imaged with an EVOS M7000 inverted fluorescence microscope (Thermo Fisher, Waltham, MA, USA).

### 5.4. Liver Function Analysis

Serum activities of ALT and AST were determined using commercial kits (Jiancheng, Nanjing, China). The values were reported as U/L.

### 5.5. Cell Culture

HepG2, a human hepatocellular carcinoma cell line, was obtained from Gefan Biotechnology (Shanghai, China). The cells were cultured in DMEM (Gibco, Grand Island, NY, USA) supplemented with 10% fetal bovine serum (FBS; TransGen Biotech, Beijing, China) and 1% penicillin–streptomycin (Solarbio, Beijing, China). The cells were maintained at 37 °C in a humidified incubator with 5% CO_2_.

### 5.6. Cell Viability Assay

HepG2 cells were cultivated in 96-well plates at 5 × 10^3^ cells/well, then exposed to AFB1 (0, 2.0, 4.0, 6.0, 8.0 or 10.0 μM) or PAT (0, 2.0, 4.0, 6.0, 8.0 or 10.0 μM) for 24 h. CCK-8 reagent was then added at 10% (*v*/*v*), and plates were incubated at 37 °C for 1–2 h before absorbance measurement at 450 nm. Six replicates were performed in each group. Concentrations that maintained cell viability above 90% were selected for subsequent experiments.

### 5.7. LDH Release Assay

The cells were cultivated in 96-well plates and divided into four groups: control, AFB1 (4 μM), PAT (4 μM), and AFB1 (4 μM) + PAT (4 μM). After 24 h treatment, culture supernatants were collected. LDH release was measured using a commercial LDH kit (Jiancheng, Nanjing, China).

### 5.8. MDA and SOD Measurements

The tissue and cell homogenates were centrifuged at 3000 rpm for 10 min at 4 °C, and the supernatants were collected for subsequent biochemical analysis. MDA and SOD activity were determined using commercial assay kits (Jiancheng, Nanjing, China).

### 5.9. Intracellular ROS Detection

The DCFH-DA fluorescent probe was used to detect intracellular ROS levels. HepG2 cells were treated with 10 μM DCFH-DA at 37 °C for 30 min in the dark, followed by washing with PBS. Fluorescence signals were recorded using an EVOS M7000 microscope (Thermo Fisher, Waltham, MA, USA), and fluorescence intensity was quantified with ImageJ software (NIH, Bethesda, MD, USA; version 1.54d).

### 5.10. ELISA for Cytokines in Culture Supernatants

The supernatants from cultured cells were harvested and centrifuged at 1000× *g* for 10 min, followed by determination of IL-1β, IL-6, and TNF-α using ELISA kits (ABclonal, Wuhan, China).

### 5.11. Z-VAD-FMK Intervention Experiment

HepG2 cells were preincubated with Z-VAD-FMK (0, 10, 20, or 30 μM) for 1 h to evaluate the involvement of apoptosis in the combined toxicity and were then exposed to AFB1 + PAT (4 μM + 4 μM) for 24 h. The working concentration was determined based on the CCK-8 results and used in the following experiments.

### 5.12. Caspase-3 Activity Assay

Following centrifugation at 16,000× *g* for 15 min at 4 °C, the supernatants from cell lysates were used for detection with a commercial caspase-3 activity assay kit (Beyotime, Shanghai, China).

### 5.13. AO/EB Staining

HepG2 cells were cultivated in 12-well plates and rinsed with PBS. Each well was treated with 5 μL of acridine orange (AO) and 5 μL of ethidium bromide (EB) staining solutions (Sangon Biotech, Shanghai, China), and the cells were then incubated in the dark for 5 min. Fluorescence images were subsequently captured with an EVOS M7000 microscope (Thermo Fisher Scientific, Waltham, MA, USA).

### 5.14. Quantitative Real-Time PCR (qPCR)

Total RNA from liver tissues and HepG2 cells was prepared with TRIzol reagent and then reverse-transcribed to obtain cDNA using the Evo M-MLV reverse transcription premix kit (Accurate, Changsha, China). qPCR was subsequently carried out with the SYBR Green Pro Taq HS premix kit (Accurate, Changsha, China) on an ABI Prism StepOne Plus system (Applied Biosystems, Foster City, CA, USA). Relative transcript levels were calculated by the 2^−ΔΔCt^ method. The primer sequences are listed in Table 1 and Table 2.

### 5.15. Western Blotting

The cells were washed with PBS and lysed in RIPA buffer containing PMSF. The BCA protein assay kit (Beyotime, Shanghai, China) was used to measure protein concentrations. Mixed with loading buffer, the protein was denatured at 95 °C for 10 min, separated by SDS-PAGE, and then transferred to PVDF membranes (Millipore, Billerica, MA, USA). After blocking with 5% non-fat milk for 2 h at room temperature, the membranes were incubated overnight at 4 °C with primary antibodies against Bax (1:2000), Bcl-2 (1:3000), and caspase-3 (1:1000), followed by HRP-conjugated secondary antibody (1:10,000) for 45 min at room temperature after three TBST washes. Protein bands were visualized using ECL reagent (Abbkine, Wuhan, China) and imaged with a chemiluminescence system (Vazyme, Nanjing, China). Band intensities were quantified with ImageJ software (NIH, Bethesda, MD, USA; version 1.54d).

### 5.16. Statistical Analysis

Data were analyzed using GraphPad Prism 10.1.2 (Boston, MA, USA) and are presented as the mean ± standard deviation (SD). For endpoints based on the four-group design (control, AFB1, PAT, and AFB1 + PAT), two-way analysis of variance (ANOVA) was used to evaluate the main effects of AFB1 and PAT and their interaction in accordance with the statistical framework for a 2 × 2 design described by Slinker [52]. For pairwise comparisons among the four groups shown in the figures, one-way ANOVA followed by Tukey’s multiple comparisons test was applied. Other experiments, including concentration-screening and inhibitor-intervention experiments, were analyzed by one-way ANOVA followed by Tukey’s multiple comparisons test. Differences were considered statistically significant when *p* < 0.05.

## Figures and Tables

**Table 1:** Primer sequences used for qPCR in mice.

Gene	RefSeq Accession Number	Primer Sequences (5′-3′)
IL-6	NM_031168.2	F: CTGGTCTTCTGGAGTACCATAGC R: GTGACTCCAGCTTATCTCTTGGT
α-SMA	XM_006526606.2	F: CCCTGAAGAGCATCCGACAC R: CCAGAGTCCAGCACAATACCA
IL-1β	NM_008361.4	F: AAGCTTCCTTGTGCAAGTGTC R: TAGCCCTCCATTCCTGAAAGC
Vimentin	NM_011701.4	F: GGCTCGTCACCTTCGTGAAT R: AGGCAGAGAAATCCTGCTCTC
TNF-α	NM_001278601.1	F: ACAGAAAGCATGATCCGCGA R: GAGGCTGAGACATAGGCACC
Bax	NM_001411994.1	F: CCAAGAAGCTGAGCGAGTGT R: ACGTCAGCAATCATCCTCTGC
Bcl-2	NM_009741.5	F: CAGGATAACGGAGGCTGGGATG R: TATGCACCCAGAGTGATGCAG
Caspase-3	NM_001284409.1	F: AGCTTGGAACGGTACGCTAAG R: CCAGAGTCCACTGACTTGCT
GAPDH	NM_001289726.2	F: TGTGTCCGTCGTGGATCTGA R: TTGCTGTTGAAGTCGCAGGAG

IL-6: interleukin-6, α-SMA: alpha-smooth muscle actin, IL-1β: interleukin-1 beta, TNF-α: tumor necrosis factor alpha, Bax: Bcl-2 associated X protein, Bcl-2: B-cell lymphoma-2, Caspase-3: cysteine-dependent aspartate-specific protease-3, GAPDH: glyceraldehyde-3-phosphate dehydrogenase.

**Table 2:** Primer sequences used for qPCR in HepG2 cells.

Gene	RefSeq Accession Number	Primer Sequences (5′-3′)
IL-6	NM_000600.5	F: CCTGAACCTTCCAAAGATGGC R: TTCACCAGGCAAGTCTCCTCA
α-SMA	NM_001141945.3	F: CAGGACCACCGGCATCGT R: AAGGAGTAGCCACGCTCAGT
IL-1β	XM_047444175.1	F: AGCCATGGCAGAAGTACCTG R: CCTGGAAGGAGCACTTCATCT
Vimentin	NM_003380.5	F: CTAACCAACGACAAAGCCCG R: ACGCATTGTCAACATCCTGT
TNF-α	NM_000594.4	F: GCTCCCCAAGAAGACAGGG R: TTCGAGAAGATGATCTGACTGCC
Bax	NM_001291428.2	F: GGTTGTCGCCCTTTTCTACTTTG R: GTCCAGCCCATGATGGTTCT
Bcl-2	NM_000633.3	F: ATGTGTGTGGAGAGCGTCAA R: CGGTTCAGGTACTCAGTCATCC
Caspase-3	NM_001354777.2	F: CATGGAAGCGAATCAATGGACT R: CTGTACCAGACCGAGATGTCA
GAPDH	NM_001256799.3	F: ACAACTTTGGTATCGTGGAAGG R: GCCATCACGCCACAGTTTC

## Data Availability

The original contributions presented in this study are included in the article. Further inquiries can be directed to the corresponding author.

## Abbreviations

The following abbreviations are used in this manuscript:

AFB1	Aflatoxin B1
PAT	Patulin
NOAEL	No-observed adverse effect level
GSH	Glutathione
ALT	Alanine aminotransferase
AST	Aspartate aminotransferase
ROS	Reactive oxygen species
MDA	Malondialdehyde
SOD	Superoxide dismutase
IL-6	Interleukin-6
IL-1β	Interleukin-1 beta
TNF-α	Tumor necrosis factor α
α-SMA	Alpha-smooth muscle actin
Bax	Bcl-2 associated X protein
Bcl-2	B-cell lymphoma-2
Caspase-3	Cysteine-dependent aspartate-specific protease-3
Z-VAD-FMK	Benzyloxycarbonyl-Val-Ala-Asp (OMe)-fluoromethylketone
HepG2	Human hepatocellular carcinoma cell line
GAPDH	Glyceraldehyde-3-phosphate dehydrogenase
LDH	Lactate dehydrogenase
